# Supplementary material for: Profiling of the Candidate Interacting Proteins of SELF-PRUNING 6A (SP6A) in Solanum tuberosum
Source: Int J Mol Sci. 2022 Aug 15;23(16):9126. doi: 10.3390/ijms23169126 (PMC9408985; doi:10.3390/ijms23169126)
Supplement: Supplementary file 1 [file ijms-23-09126-s001.zip › ijms-1828488-supplementary.pdf]

Supplementary Material

Profiling of the SELF-PRUNING 6A (SP6A) candidate interacting proteins in *Solanum tuberosum*

Enshuang Wang, Tengfei Liu, Xiaomeng Sun, Shenglin Jing, Tingting Zhou, Tiantian Liu, Botao Song

Figure S1 Identification of CFU and insertion fragments from the secondary library.

Figure S2 Validate the interaction between StSP6A and the interactors by yeast two-hybrid assay.

Figure S3 Subcellular localization of StSP6A

Figure S4 Promoter analyses of *StGA2ox.*

Supplementary Table S1 Putative interaction proteins of StSP6A identified from the Y2H library

Supplementary Table S2 A list of primers used in this study

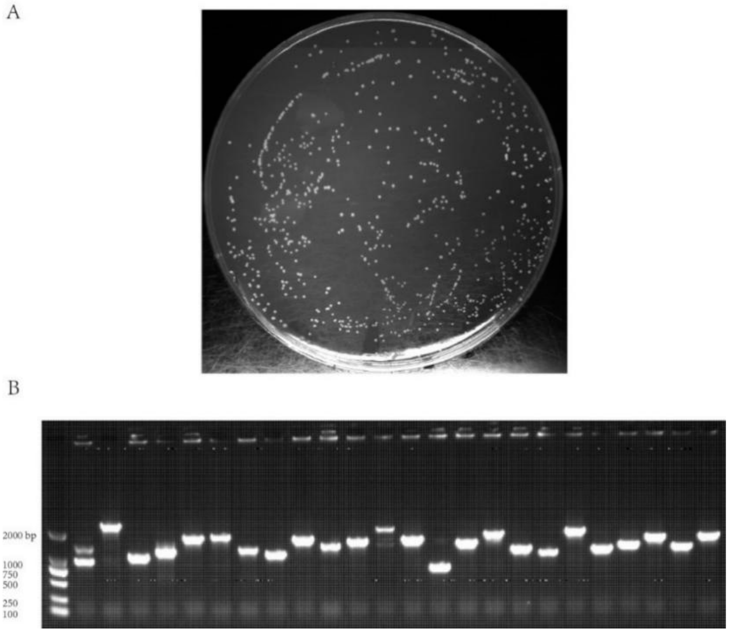

Figure S1 Identification of CFU and insertion fragments from the secondary library

A: Identification of library cell density. B: Identification of insertion fragments by PCR (Lane 2-25) and Marker DL2000 (Lane 1).

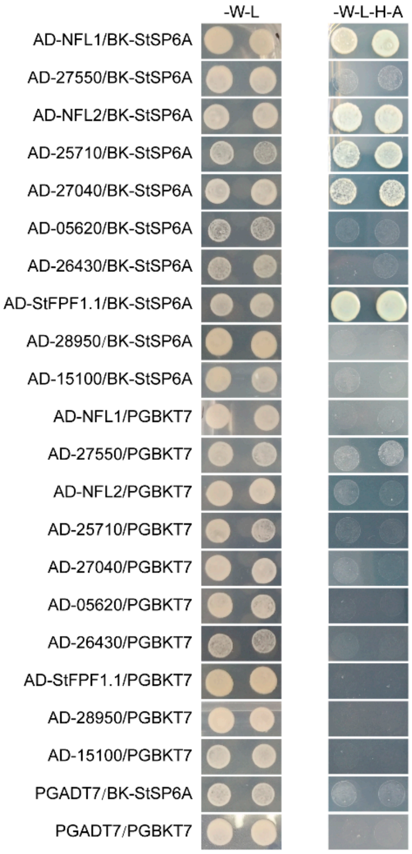

Figure S2 Validate the interaction between StSP6A and the interactors by yeast two-hybrid assay. Ten interactors were selected. The BK-StSP6A and its interactors were co-expressed in yeast strain AH109. -W-L (medium without tryptophan and leucine); -W-L-H-A (medium without tryptophan, leucine, histidine and adenine). BK (pGBKT7) and AD (pGADT7) are the bait and prey vectors, respectively.

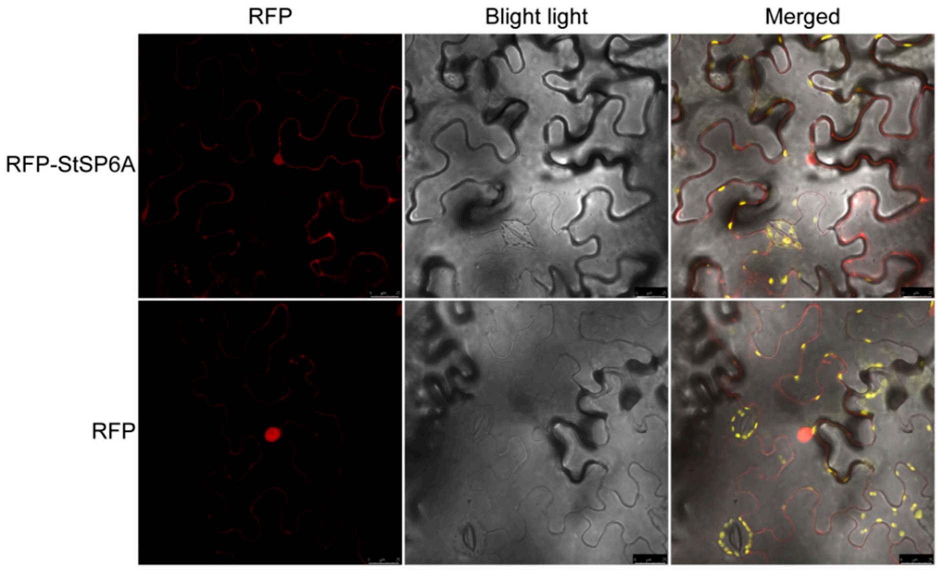

Figure S3 Subcellular localization of StSP6A

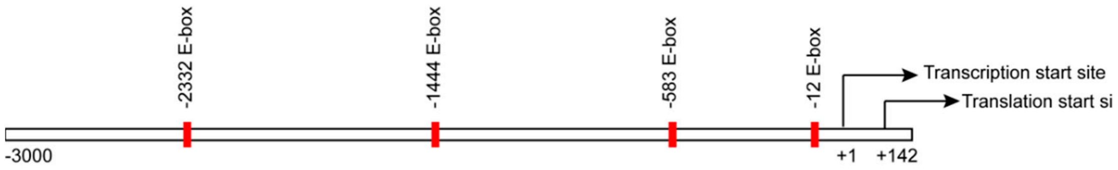

Figure S4 Promoter analyses of *StGA2ox.* 3000 bp fragments of the *StGA2ox* promoter were selected for analyzing.

Supplementary Table S1 Putative interaction proteins of StSP6A identified from the Y2H library

Locus name	Annotation	frequency
Soltu.DM.08G022890.1	2Fe-2S ferredoxin-like superfamily protein	1
Soltu.DM.06G027300.2	40s ribosomal protein SA	1
Soltu.DM.12G000940.1	5'-AMP-activated protein kinase beta-2 subunit protein	1
Soltu.DM.07G005190.1	60S acidic ribosomal protein family	2
Soltu.DM.05G000980.3	6-phosphogluconolactonase	1
Soltu.DM.06G015100.1	Acyl-CoA N-acyltransferases （NAT） superfamily protein	2
Soltu.DM.03G000330.1	ATP sulfurylase	1
Soltu.DM.09G007020.1	basic pathogenesis-related protein	1
Soltu.DM.02G026970.1	basic HLH protein	2
Soltu.DM.02G007350.1	basic HLH protein	1
Soltu.DM.10G027550.1	beta-1,3-glucanase	1
Soltu.DM.01G045950.1	CAP （Cysteine-rich secretory proteins） superfamily protein	1
Soltu.DM.12G000130.1	chromomethylase	1
Soltu.DM.01G042420.2	clathrin adaptor complexes medium subunit family protein	1
Soltu.DM.09G001860.1	Concanavalin A-like lectin protein kinase family protein	1
Soltu.DM.02G025390.1	conserved hypothetical protein	1
Soltu.DM.03G003130.1	conserved hypothetical protein	1
Soltu.DM.09G019380.1	conserved hypothetical protein	1
Soltu.DM.04G037140.1	Cupredoxin superfamily protein	6
Soltu.DM.08G027040.1	cytochrome P450, family 76, subfamily C, polypeptide	1
Soltu.DM.02G034200.2	DCD （Development and Cell Death） domain protein	1
Soltu.DM.11G022820.1	DNA-directed RNA polymerase, RBP11-like	2
Soltu.DM.05G025710.1	DNAJ homologue	1
Soltu.DM.01G051590.2	expansin-like A1	1
Soltu.DM.10G025630.2	FASCICLIN-like arabinogalactan protein 17 precursor	2
Soltu.DM.12G024780.1	fatty acyl-ACP thioesterases B	1
Soltu.DM.01G021950.1	flowering promoting factor	1
Soltu.DM.06G000550.1	glutathione S-transferase phi	2
Soltu.DM.12G005020.1	glutathione S-transferase phi	1
Soltu.DM.01G041440.1	glutathione S-transferase zeta	1
Soltu.DM.12G004790.1	glyceraldehyde-3-phosphate dehydrogenase B subunit	1
Soltu.DM.03G024400.1	glyceraldehyde-3-phosphate dehydrogenase C subunit	1
Soltu.DM.05G010790.1	glyceraldehyde-3-phosphate dehydrogenase C2	1
Soltu.DM.12G005890.1	Granulin repeat cysteine protease family protein	1
Soltu.DM.06G005620.1	GTP binding Elongation factor Tu family protein	1
Soltu.DM.04G035870.2	GTP-binding protein, HflX	1
Soltu.DM.01G024990.1	HIT zinc finger ;PAPA-1-like conserved region	1
Soltu.DM.04G026490.1	hypothetical protein	6
Soltu.DM.08G028950.1	hypothetical protein	1
Soltu.DM.06G031500.1	metallothionein 2A	1
Soltu.DM.09G027700.1	MLP-like protein	1
Soltu.DM.04G036070.1	Papain family cysteine protease	1
Soltu.DM.03G014310.1	peptide met sulfoxide reductase	1
Soltu.DM.04G024830.1	phosphoribosyl-aminoimidazole synthetase （PUR5）	2
Soltu.DM.12G022950.1	photosynthetic electron transfer C	2
Soltu.DM.05G026770.1	photosystem I light harvesting complex gene	1

Locus name	Annotation	frequency
Soltu.DM.02G001740.1	photosystem II stability, chloroplast （HCF136）	1
Soltu.DM.04G021720.1	Pollen Ole e 1 allergen and extensin family protein	1
Soltu.DM.07G025540.1	polyubiquitin	5
Soltu.DM.04G004650.3	Pyruvate kinase family protein	1
Soltu.DM.06G024950.1	receptor for activated C kinase 1B	1
Soltu.DM.04G006090.1	Ribosomal protein L10 family protein	1
Soltu.DM.06G034170.1	Ribosomal protein L14p/L23e family protein	1
Soltu.DM.06G002880.1	Ribosomal protein S4 （RPS4A） family protein	1
Soltu.DM.09G016990.1	Ribosomal protein S8e family protein	1
Soltu.DM.02G025840.2	Ribulose bisphosphate carboxylase family protein	4
Soltu.DM.02G025810.1	Ribulose bisphosphate carboxylase family protein	4
Soltu.DM.02G007030.4	Ribulose bisphosphate carboxylase family protein	1
Soltu.DM.03G004480.1	Ribulose bisphosphate carboxylase family protein	1
Soltu.DM.11G016190.1	Rieske （2Fe-2S） domain-containing protein	1
Soltu.DM.04G025730.2	root cap 1 （RCP1）	1
Soltu.DM.09G029630.1	S-adenosyl-L-homocysteine hydrolase	1
Soltu.DM.04G025070.2	S-adenosyl-L-methionine-dependent methyltransferases superfamily protein	2
Soltu.DM.03G017000.1	S-adenosyl-L-methionine-dependent methyltransferases superfamily protein	1
Soltu.DM.01G040830.1	S-adenosylmethionine synthetase	1
Soltu.DM.07G026830.1	SHV3-like	2
Soltu.DM.08G026430.1	Subtilase family protein	2
Soltu.DM.03G037270.1	Subtilase family protein	1
Soltu.DM.06G003240.2	thiaminC	1
Soltu.DM.12G029650.1	thioredoxin M-type	1
Soltu.DM.06G027910.5	Transketolase family protein	2
Soltu.DM.10G019950.1	tubulin beta	1
Soltu.DM.11G015010.1	Ubiquinol-cytochrome C reductase iron-sulfur subunit	1
Soltu.DM.02G027730.3	voltage dependent anion channel	2
Soltu.DM.03G008530.1	voltage dependent anion channel	2
Soltu.DM.02G009970.1	voltage dependent anion channel	3
Soltu.DM.04G037150.1	Zim17-type zinc finger protein	1

Supplementary Table S2. A list of primers used in this study

Primer name	Sequence(5'-3')	Target gene	Amplicon (bp)	Vector	Usage
BK-SP6A-EcoRI-F	ATGGCCATGGAGGCCGAATTCATGCC TAGAGTTGATCCATTGAT	Soltu.DM.0 5G026370.1	564	pGBKT7	Y2H assay
BK-SP6A-SalI-R	ATGCGGCCGCTGCAGGTCGACTTATG CGCGACGTCCTCC	Soltu.DM.0 5G026370.1	564	pGBKT7	Y2H assay
AD-FPF1.1-EcoRI-F	ATGGAGGCCAGTGAATTCATGTCTG GTGTTTGGGTATTC	Soltu.DM.0 1G021950.1	321	pGADT7	Y2H assay
AD-FPF1.1-BamHI-R	CTCGAGCTCGATGGATCCCTACATGT CCCTAACTTCAAAC	Soltu.DM.0 1G021950.1	321	pGADT7	Y2H assay
AD-NFL1-EcoRI-F	ATGGAGGCCAGTGAATTCATGGAGC TCACTCAACAAGATT	Soltu.DM.0 2G026970.1	1008	pGADT7	Y2H assay
AD-NFL1-BamHI-R	CTCGAGCTCGATGGATCCTCATAGA CATCTTCCTCCATAA	Soltu.DM.0 2G026970.1	1008	pGADT7	Y2H assay
AD-NFL2-EcoRI-F	ATGGAGGCCAGTGAATTCATGGAGC TTAGTGAACATGATAT	Soltu.DM.0 2G007350.1	981	pGADT7	Y2H assay
AD-NFL2-BamHI-R	CTCGAGCTCGATGGATCCCTAAAGA CATCTTCCTCCAAAGC	Soltu.DM.0 2G007350.1	981	pGADT7	Y2H assay
AD-25710-EcoRI-F	ATGGAGGCCAGTGAATTCATGTTTG GGAGGGCACCGAAGA	Soltu.DM.0 5G025710.1	1260	pGADT7	Y2H assay
AD-25710-BamHI-R	CTCGAGCTCGATGGATCCTTACTGTT GTGCACATTGAACTC	Soltu.DM.0 5G025710.1	1260	pGADT7	Y2H assay
AD-27040-EcoRI-F	ATGGAGGCCAGTGAATTCATGGCAC AGCAATTTGAAATTT	Soltu.DM.0 8G027040.1	1488	pGADT7	Y2H assay
AD-27040-BamHI-R	CTCGAGCTCGATGGATCCTCATTTTC TTGCCTTGGGAATC	Soltu.DM.0 8G027040.1	1488	pGADT7	Y2H assay
AD-28950-EcoRI-F	ATGGAGGCCAGTGAATTCATGTCTTC AATGCAATGCTACA	Soltu.DM.0 8G028950.1	624	pGADT7	Y2H assay
AD-28950-BamHI-R	CTCGAGCTCGATGGATCCTTAGCAAT GACGCTCGTCATCG	Soltu.DM.0 8G028950.1	624	pGADT7	Y2H assay
AD-27550-EcoRI-F	ATGGAGGCCAGTGAATTCATGGCTT GTACCAAACTACATT	Soltu.DM.1 0G027550.1	1035	pGADT7	Y2H assay
AD-27550-BamHI-R	CTCGAGCTCGATGGATCCCTAGTTGA AACTGATCGCGTAT	Soltu.DM.1 0G027550.1	1035	pGADT7	Y2H assay
AD-05620-EcoRI-F	ATGGAGGCCAGTGAATTCATGGGTA AGGAGAAGATTCACA	Soltu.DM.0 6G005620.1	1347	pGADT7	Y2H assay
AD-05620-BamHI-R	CTCGAGCTCGATGGATCCTCACTTTC CCTTCTTCTGGGCA	Soltu.DM.0 6G005620.1	1347	pGADT7	Y2H assay
AD-26430-EcoRI-F	ATGGAGGCCAGTGAATTCATGGGAT TCTTGAAAATC	Soltu.DM.0 8G026430.1	2238	pGADT7	Y2H assay
AD-26430-BamHI-R	CTCGAGCTCGATGGATCCCTAGGCT AACACAACTGCAA	Soltu.DM.0 8G026430.1	2238	pGADT7	Y2H assay
AD-15100-EcoRI-F	ATGGAGGCCAGTGAATTCATGCAGA TCTTTGTGAAAACTC	Soltu.DM.0 6G015100.1	1374	pGADT7	Y2H assay
AD-15100-BamHI-R	CTCGAGCTCGATGGATCCTTAAAAA CCACCACGGAGACGG	Soltu.DM.0 6G015100.1	1374	pGADT7	Y2H assay
CYFP-NFL1-BamHI-F	TTCCAGATTACGCTGGATCCATGGAG CTCACTCAACAAGATT	Soltu.DM.0 2G026970.1	1008	CYFP	BiFC
CYFP-NFL1-SalI-R	CCGAATTCACTAGTGTCGACTCATAG ACATCTTCCTCCATAA	Soltu.DM.0 2G026970.1	1008	CYFP	BiFC
CYFP-NFL2-BamHI-F	TTCCAGATTACGCTGGATCCATGGAG CTTAGTGAACATGATAT	Soltu.DM.0 2G007350.1	981	CYFP	BiFC

CYFP-NFL2-SalI-R	CCGAATTCACTAGTGTCGACCTAAAG ACATCTTCCTCCAAAGC	Soltu.DM.0 2G007350.1	981	CYFP	BiFC
CYFP-FPF1.1-BamHI-F	TTCCAGATTACGCTGGATCCATGTCT GGTGTTTGGGTATTC	Soltu.DM.0 1G021950.1	321	CYFP	BiFC
CYFP-FPF1.1-SalI-R	CCGAATTCACTAGTGTCGACCTACAT GTCCCTAACTTCAAAC	Soltu.DM.0 1G021950.1	321	CYFP	BiFC
NYFP-SP6A-BamHI-F	CTGAGGAGGATCTTGGATCCATGCCT AGAGTTGATCCATTGAT	Soltu.DM.0 5G026370.1	561	NYFP	BiFC
NYFP-SP6A-SalI-R	CCGAATTCACTAGTGTCGACTTATGC GCGACGTCCTCC	Soltu.DM.0 5G026370.1	561	NYFP	BiFC
PB7-NFL1-Bsp1407I-F	CTCGGCATGGACGAGCTGTACAAGAT GGAGCTCACTCAACAAGAT	Soltu.DM.0 2G026970.1	1008	PB7GWF 2	Subcellular localization
PB7-NFL1-Bsp1407I-R	CGGGATATCACCACTTTGTACATTATC ATAGACATCTTCCTCCATAA	Soltu.DM.0 2G026970.1	1008	PB7GWF 2	Subcellular localization
PB7-NFL2-Bsp1407I-F	CTCGGCATGGACGAGCTGTACAAGAT GGAGCTTAGTGAACATGATAT	Soltu.DM.0 2G007350.1	981	PB7GWF 2	Subcellular localization
PB7-NFL2-Bsp1407I-R	CGGGATATCACCACTTTGTACATTACT AAAGACATCTTCCTCCAAAGC	Soltu.DM.0 2G007350.1	981	PB7GWF 2	Subcellular localization
PB7-FPF1.1-Bsp1407I-F	CTCGGCATGGACGAGCTGTACAAGAT GTCTGGTGTTTGGGTATTC	Soltu.DM.0 1G021950.1	321	PB7GWF 2	Subcellular localization
PB7-FPF1.1-Bsp1407I-R	CGGGATATCACCACTTTGTACATTACT ACATGTCCCTAACTTCAAAC	Soltu.DM.0 1G021950.1	321	PB7GWF 2	Subcellular localization
SP6A-RFP-SpeI-F	CCTGCAGGCGGCCGCACTAGTATGC CTAGAGTTGATCCATTGAT	Soltu.DM.0 5G026370.1	561	PB7WGR 2	Subcellular localization
SP6A-RFP-SpeI-R	GACGTCCTCGGAGGAGGCCATTGCG CGACGTCCTCCA	Soltu.DM.0 5G026370.1	561	PB7WGR 2	Subcellular localization
FPF_q_rev	CTAGTTGAGAACGCCGGTGACT	Soltu.DM.0 1G021950.1	127		qPCR
FPF_q_for	CCCATCCAAGAGAGTACAAATTCC	Soltu.DM.0 1G021950.1	127		qPCR
SP6A for	GACGATCTTCGCAACTTTTACA	Soltu.DM.0 5G026370.1	75		qPCR
SP6A rev	CCTCAAGTTAGGGTCGCTTG	Soltu.DM.0 5G026370.1	75		qPCR
qRT-NFL1-F	AGCATTAGGGCTTGAGGTGC	Soltu.DM.0 2G026970.1	128		qPCR
qRT-NFL1-R	ACAGAGCTTGCTTCACGTCT	Soltu.DM.0 2G026970.1	128		qPCR
qRT-NFL2-F	GCAAAGCCAGGGTTGTTGTT	Soltu.DM.0 2G007350.1	129		qPCR
qRT-NFL2-R	ATGCTCCCTTGCCTCAGAAC	Soltu.DM.0 2G007350.1	129		qPCR
RTef1α-F	ATTGGAAACGGATATGCTCCA	Soltu.DM.0 6G005620.1	101		qPCR
RTef1α-R	TCCTTACCTGAACGCCTGTCA	Soltu.DM.0 6G005620.1	101		qPCR
